# Developmental roadmap for antimicrobial susceptibility testing systems

**DOI:** 10.1038/s41579-018-0098-9

**Published:** 2018-10-17

**Authors:** Alex van Belkum, Till T. Bachmann, Gerd Lüdke, Jan Gorm Lisby, Gunnar Kahlmeter, Allan Mohess, Karsten Becker, John P. Hays, Neil Woodford, Konstantinos Mitsakakis, Jacob Moran-Gilad, Jordi Vila, Harald Peter, John H. Rex, Wm. Michael Dunne, the JPIAMR AMR-RDT Working Group on Antimicrobial Resistance and Rapid Diagnostic Testing

**Affiliations:** 1 bioMérieux, Data Analytics Unit, La Balme Les Grottes, France; 2 The University of Edinburgh, Edinburgh Medical School, Division of Infection and Pathway Medicine, The Chancellor’s Building, Edinburgh, UK; 3 Curetis GmbH, Holzgerlingen, Germany; 4 University of Copenhagen, Amager and Hvidovre Hospital, Department of Clinical Microbiology, Hvidovre, Denmark; 5 EUCAST Development Laboratory for Antimicrobial Susceptibility Testing, c/o Clinical Microbiology, Central Hospital, Växjö, Sweden; 6 bioMérieux, IT Solutions and Services, Durham, NC USA; 7 University of Münster, Institute of Medical Microbiology, Münster, Germany; 8 Department of Medical Microbiology & Infectious Diseases, Erasmus University Medical Centre (Erasmus MC), Rotterdam, Netherlands; 9 NIS Laboratories, National Infection Service, Public Health England, London, UK; 10 Hahn-Schickard, Freiburg, Germany; 11 Department of Health Policy and Management, School of Public Health, Ben-Gurion University of the Negev, Beer-Sheva, Israel; 12 ESCMID Study Group for Genomic and Molecular Diagnostics (ESGMD), Basel, Switzerland; 13 University of Barcelona, School of Medicine, Clinical Microbiology, Hospital Clinic IS Global, 08036 Barcelona, Spain; 14 Fraunhofer IZI-BB, Potsdam, Germany; 15 F2G Ltd, Lankro Way, Eccles, Manchester, UK; 16 bioMérieux, Data Analytics Unit, 100 Rodolphe Street, Durham, NC USA

**Keywords:** Infectious-disease diagnostics, Health care

## Abstract

Antimicrobial susceptibility testing (AST) technologies help to accelerate the initiation of targeted antimicrobial therapy for patients with infections and could potentially extend the lifespan of current narrow-spectrum antimicrobials. Although conceptually new and rapid AST technologies have been described, including new phenotyping methods, digital imaging and genomic approaches, there is no single major, or broadly accepted, technological breakthrough that leads the field of rapid AST platform development. This might be owing to several barriers that prevent the timely development and implementation of novel and rapid AST platforms in health-care settings. In this Consensus Statement, we explore such barriers, which include the utility of new methods, the complex process of validating new technology against reference methods beyond the proof-of-concept phase, the legal and regulatory landscapes, costs, the uptake of new tools, reagent stability, optimization of target product profiles, difficulties conducting clinical trials and issues relating to quality and quality control, and present possible solutions.

## Abstract (web summary)

This Consensus Statement presents the barriers that currently prevent the timely development and implementation of novel and rapid antimicrobial susceptibility testing platforms, including the costs involved, uptake of new tools, legal and regulatory aspects, difficulties conducting clinical trials and quality control, and presents possible solutions.

## Introduction

Antimicrobial resistance (AMR) is a steadily increasing global problem, and drug-resistant pathogens kill at least 25,000 infected people annually in the European Union alone^1^ (Box 1). The development of AMR is limiting the number of antibiotics that can be used to successfully treat infections, especially if the empirical prescription of antibiotics is necessary. The European Centre for Disease Prevention and Control (ECDC) has estimated that to date 30–50% of all antimicrobials prescribed to human patients are unnecessary^2^, and over-prescription of antimicrobials further promotes the development and spread of resistance.

Antimicrobial susceptibility testing (AST) aims to ensure that suitable antibiotics are prescribed and to monitor the selection and emergence of resistant pathogens in infected individuals. Information on local patterns of antimicrobial susceptibility can be collected using AST, so that policies guiding the empiric choice of therapy can be based on current data on local resistance trends (also known as the local or institutional antibiogram). AST can also help to identify isolates with defined resistance mechanisms of major interest to infection prevention and control (for example, extended-spectrum β-lactamase producers, carbapenemase-producing Enterobacteriaceae, methicillin-resistant *Staphylococcus aureus* (MRSA) and vancomycin-resistant enterococci). Furthermore, AST is key for the assessment of resistance incidence and prevalence in epidemiological studies that examine the origin and spread of resistance, including studies on the effectiveness of measures taken to counteract spread.

**Fig. 1:**
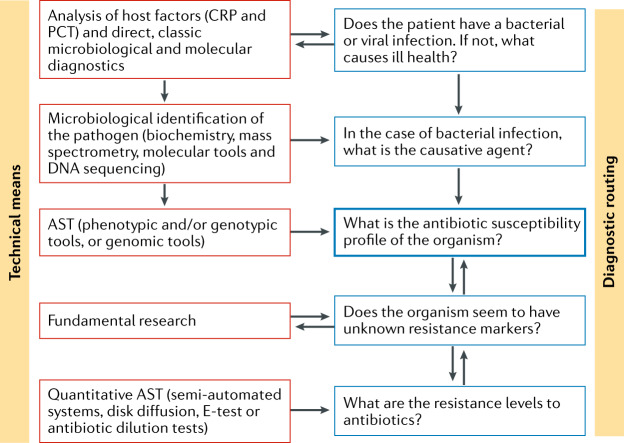
Triaging infections using diagnostic testing. Various technical means are available for identifying the causative agents of microbial infections and for generating antibiotic susceptibility profiles, which can inform the suitable course of treatment (diagnostic routing). First, host factors, such as C-reactive protein (CRP), procalcitonin (PCT) and others are analysed to, for instance, distinguish viral from bacterial infection. Following the identification of the causative bacterial pathogen, antimicrobial susceptibility testing (AST) is the next step (except in some specific molecular tests) to generate an antibiotic susceptibility profile. AST ensures that suitable antibiotics are prescribed to a patient. Beyond the outlined routine diagnostic process, there is opportunity to follow up with fundamental research to analyse the presence of novel resistance markers (possibly identifying novel resistance mechanisms) and to determine the resistance levels to the antibiotic.

Several different technical means are available for identifying the causative agents of microbial infections and deciding on a suitable course of treatment at different stages of the diagnostic pipeline (Fig. 1). However, in terms of actually facilitating targeted antimicrobial therapy, it is important to note that some clinical microbiology laboratories in different global geographical regions may not have access to the currently most popular and commercially available AST platforms. These platforms include several semi-automated systems and manual tests such as, for example, the application of antibiotic gradient strips and disk diffusion methodology. As a technique, AST infers the concentration of an antibiotic that is required to inhibit multiplication of a microorganism in vitro and that would be achievable in patients. This can be accomplished via growth-based (phenotypic) or molecular (genotypic) methods. It is noteworthy that genotypic methods mostly detect resistance factors, whereas phenotypic methods enable real susceptibility testing. Phenotypic AST detects the arrest of bacterial cell growth in the presence of static or cidal antimicrobial agents^3^. Genotypic AST attempts to identify specific resistance genes or genetic mutations using molecular or genomic (usually DNA-based, amplification-based or sequencing-based) methods. Genotypic methods are surrogates for AST, and susceptibility has to be validated with phenotypic tests. However, genotypic testing is very useful and fast (for example, testing for the presence of the resistance determinants *mecA* and *mecC* for the identification of MRSA can be performed in minutes). Conversely, phenotypic AST may not always accurately reflect the underlying genotype of a microorganism, and therefore, additional phenotypic testing may be required. Finally, local epidemiology may greatly affect test performance and utility as, for instance, increased prevalence of hard-to-detect resistance traits may skew test performance.

In current clinical microbiology laboratories, AST is usually performed after a bacterial infectious agent has been cultured and identified at the species level. However, AST is time consuming, as it involves regrowth of the organism in the absence and presence of the relevant antibiotics. Qualified laboratory scientists who are proficient in the use of AST may not always be available during a particular shift during the day. Often, data are not available until the end of a full test run or final validation of the complete data set. It is crucial to improve laboratory procedures and staffing hours for the clinical laboratory to ensure effective testing. Furthermore, current AST turnaround times are usually between 12 h and 48 h. So-called rapid testing, defined as being feasible within an 8-h working shift, supports antibiotic stewardship programmes and promotes the prudent use of antimicrobials^4^. Although the AST platforms that are currently most used are robust and represent added value to the clinical diagnostic microbiology laboratory, their main shortcoming is a somewhat long time to result (TTR) and a lack of full automation, which may hinder the accurate prescription of antibiotics. From a basic microbiological perspective, it has to be realized that our changing understanding of current antibiotic resistance mechanisms, the discovery of new mechanisms, epidemiological aspects, variation of the growth-associated lag time, heterogeneity of resistance and the occasional need for pre-diagnostic induction of resistance may all provide important barriers when applying AST. Therefore, to facilitate targeted (and personalized) antimicrobial prescribing practices and to help reduce the increasing global burden of antibiotic resistance, there is an urgent need for the development and implementation of novel and truly rapid AST platforms (that is, results being available in 30 min to 1 h)^5^.

Developments in the field of rapid AST platforms have been slow over the past decade^6,7^. This can be owing to suboptimal sensitivity and specificity, somewhat high purchase and testing costs and the lack of rapid result reporting for the care-giving physicians. Moreover, the development and implementation of new AST platforms may be slowed by other factors, including considerations relating to the actual number of antibiotic targets to be included in the new platform, post-developmental validation in laboratory and clinical settings, geographical and institutional differences in the optimal antibiotic target menu, issues regarding legal and intellectual property (IP) aspects, cost-effectiveness, acceptance of new AST by end users, regulatory approval and the need for local expertise. In addition, very major errors (that is, false antibiotic susceptibility) and major errors (that is, false resistance) are a constant cause of concern. The European Committee of Antimicrobial Susceptibility Testing (EUCAST) and the Clinical and Laboratory Standards Institute (CLSI) are vigilant in regard to identifying these shortcomings. The European Commission aims to standardize all innovations in the field of rapid AST, which is also being promoted by the European Food Safety Authority (EFSA), the European Medicines Agency (EMA) and the ECDC.

Perhaps the most important factor for consideration is the TTR, as many clinical studies have shown that a delay in adequate antibiotic treatment for severe infections increases mortality^8^. TTR may be particularly important when the health-care focus is on the rapid diagnosis and treatment of antimicrobial-resistant pathogens in settings outside of routine hospital-based care and where multidrug-resistant organisms are likely to be present, for example, at a field hospital or in low-resource settings. These issues may be less important when the focus of AST results is on infection prevention and outbreak monitoring. In the latter case, several highly weighted factors that affect the development and implementation of novel AST platforms^9^ could include the comparative analysis of the effect of the implementation of new AST systems on laboratory efficiency and data quality, adequate communication between laboratory data systems and decision-support tools, and surveillance tools for historical and current diagnostic information for infection prevention and the monitoring of local outbreaks of antibiotic-resistant pathogens. Preferably, efforts should be system agnostic and applicable to both existing and future AST platforms.

It should be noted that most clinical microbiologists are not yet ready to accept the implementation of AST-only systems, given the importance of establishing the identity of microbial species in the context of clinical decision making. According to EUCAST and CLSI guidelines, identification of pathogens at the species level is currently an essential element in interpreting minimum inhibitory concentrations (MICs) of antibiotics for particular pathogens. A paradigm shift will first be needed if physicians are to base their clinical decision making on AST-only platforms^10^. By contrast, it could be advisable to integrate AST platforms with a capacity for (limited) microbial identification in any new development process. MALDI-TOF mass spectrometry (MALDI-TOF-MS) may be well suited for this purpose.

There are also potential downsides to advocating the development of more rapid AST platforms. Major concerns would include lowered sensitivity and specificity as a consequence of abbreviated clinical validation studies or inadequate testing of low prevalence markers. Polymicrobial clinical samples could also influence test accuracy and, for instance, in molecular testing, associating the right pathogen with the correct antibiotic resistance gene is crucial. Both laboratory personnel and clinicians are increasingly exposed to complicated information packages, and the correct management of such entities should result in more clinically actionable data. This suggests that large cooperative studies will be needed to guarantee an appropriate balance between the challenges listed above.

In this Consensus Statement, we present the barriers that are currently preventing the timely development and implementation of novel and rapid AST platforms, including the costs involved, uptake of new tools, legal and regulatory aspects, optimization of target product profiles, difficulties conducting clinical trials and issues with quality and quality control. This Consensus Statement was developed following discussions on the current barriers to the implementation of new antimicrobial resistance testing formats and was facilitated by the Joint Programming Initiative on Antimicrobial Resistance (JPIAMR) Working Group on Rapid Diagnostic Testing (Supplementary Table 1).

Box 1 Combating antimicrobial resistance* To tackle the spread of antimicrobial resistance (AMR) in humans, animals and the environment, we aim to have implemented national action plans, based on a One-Health approach, well underway by the end of 2018. We will promote prudent antibiotic use and strive to restrict therapeutic use. Responsible use of antibiotics in food-producing animals does not target growth promotion. Treatments should be available through prescription only. We will strengthen public awareness, infection prevention and control and improve the understanding of the issue of antimicrobials in the environment. We will promote access to affordable and quality antimicrobials, vaccines and diagnostics. We highlight the importance of fostering research and development, in particular, for priority pathogens as identified by the WHO. We call for a new international Research and Development Collaboration Hub to maximize the impact of existing and new antimicrobial basic and clinical research initiatives as well as product development.

*The authors’ version of the statement by the European Commission on the global antimicrobial resistance problem, issued during the G20 meeting in Hamburg, Germany (G20 Leaders’ Declaration: Shaping an interconnected world, published 8th July 2017).

## Needs and barriers

Existing AMR traits are spreading globally, and resistance to newly licensed antimicrobials or novel mechanisms of resistance to older agents continue to emerge^11,12^. Many high-quality and recent scientific, economical, public health-oriented and educational reports have been published on the subject^13,14^. Although these reports cover various AST-related subjects, with respect to AST, there are two basic diagnostic needs. First, there is the need for physicians to rapidly identify antibiotics that can be used to successfully treat patients infected with bacterial pathogens. Second, there is a need for epidemiological assessment, that is, detecting phenotypic resistance mechanisms and monitoring their spread. As such, AST generates surveillance data and helps to design strategic actions to control AMR dissemination. These needs go hand-in-hand with the requirement to overcome diagnostic device implementation barriers (Table 1), and in this respect none of the new AST platforms that are currently being developed (including the point-of-care (POC) tests, Box 2) are at the same level of clinical acceptance as classic routine AST methods. Although many of the newly proposed technologies show promising fields of application and good data, their current developmental status still prohibits direct clinical use. The major clinical needs identified in this section are aligned with the capacities of the current routine-applicable AST systems. For many of the newly suggested technologies, there are features that may still frustrate those clinical requirements.

**Table 1:** Barriers to the development and implementation of improved AST systems^a^

Barriers	Effect on patient	Effect on user	Effect on manufacturer	Effect on policy maker
Competence of laboratory personnel		x		
Distance between clinicians and diagnosticians		x		
High cost of test		x		
Continuous availability in the laboratory		x		
Lack of clinical outcome studies		x		
Costs of clinical studies			x	
Costs of development			x	
Data management and poor electronic medical records			x	
Finding partners (clinicians, laboratory directors, researchers, etc.) for clinical studies			x	
Intellectual property challenges associated with multiple innovation			x	
Laboratory decentralization and unlinked stewardship teams			x	
Lack of appropriate marketing			x	
Lack of pre-market commitment			x	
Quality and availability of primary materials			x	
Policy makers not understanding the development process				x
Issues concerning sample taking	x	x		
Specimen accessibility	x	x		
Knowledge gaps of physicians or clinicians in the relevance of antimicrobial resistance		x	x	
Lack of funding		x	x	
Storage, transport and stability of the test		x	x	
Sample transport and other logistical issues		x	x	
Supply chain failure		x	x	
Limited exchange between diagnostic and pharmaceutical companies			x	x
Lack of speed	x	x	x	
Test availability	x	x	x	
FDA validation and European certification (CE) including differences between countries		x	x	x
Biological hazard		x	x	x
Ethical aspects		x	x	x
Insufficient exchange between the public and private sector		x	x	x
Environmental aspects	x	x	x	x
Health practice behaviour	x	x	x	x
Communication (or lack thereof)	x	x	x	x
Lack of support programmes	x	x	x	x

^a^The table is partially based on a prior key paper in this field^81^.

Box 2 Point-of-care antimicrobial susceptibility testing Antimicrobial susceptibility testing (AST) requires specific laboratory environments and instruments, and the availability of point-of-care (POC), point-of-need or point-of-impact AST is rare^69^. The exceptions are mainly DNA tests, which are capable of detecting resistance genes at all taxonomic levels, but still, they often require phenotypic verification^32,70–73^. This is unfortunate, as POC AST would enable the rapid and personalized treatment of infections, enabling clinicians to discern, for example, between *Neisseria gonorrhoeae* strains that are susceptible to currently frequently used antibiotics or require last-line antimicrobials^25,74^. POC AST could facilitate rapid diagnosis and treatment of major infectious diseases, such as tuberculosis, in low-income and middle-income countries^75,76^. A recent study^77^ described the use of decentralized rapid culture and an associated slow AST platform for the diagnosis and susceptibility testing of samples from patients suffering from urinary tract infections, which, although not fail-proof, could function well in remote regions. Importantly, the price per test and the costs of the equipment will be major determinants for the implementation of POC AST. Compliance of POC AST with WHO ASSURED (affordable, sensitive, specific, user-friendly, rapid and robust, equipment free, deliverable to users^78^) criteria, which is especially relevant for low-income and middle-income countries, is to be hoped for but not expected soon^79^. In fact, all of the ASSURED criteria (although designed to ensure maximum implementation of POC testing in the target market) pose barriers to the development of POC AST^80^. POC tests need to be addressed individually to define their usefulness.

### Intellectual property and data protection

IP is a major driver in defining company policy. It defines competitive advantages in the market on the basis of specific knowledge and expertise developed internally within companies by experimental and literature-based studies; such studies may result in new perspectives and practical inventions. Per definition, an invention is a creative technical solution to a technical problem that cannot be easily derived from the current state of the art and covers products (for example, devices, compositions and molecules), methods and the use of methods^15^. Examples also include automation or methods of barcoding, the aesthetics of a design, an original artistic form or a distinctive sign. Inventions can be developed into trademarks, designs, copyrights and patents. Patents on inventions should be filed with an IP office and may be awarded after examination. The maximum life of a patent is 20 years, and the patent may be valid for only specific geographical areas. Patent protection is costly and depends on the geographical area that needs to be covered. Licensing of patents may reimburse some of the costs involved. However, in some cases, applying for a patent may not be advisable because trade secrets can be better protected by confidentiality.

In the field of informatics and big data, protection issues are complicated^16^. Databases of AMR gene variants are almost impossible to protect by IP. Although considerable investments have been made to establish the data sets^17^, many of these databases are freely available online for academic use (for example, the Comprehensive Antibiotic Resistance Database)^18^. Of note, ongoing, large European Union-sponsored programmes are striving to generate high-quality, open access databases, with a focus on AST relating to the use of bacterial genome databases^19–21^. It remains to be seen whether the quality and curation of the databases will remain unaffected upon termination of such (grant-funded) programmes.

Management of IP and databases can pose barriers to the development of new AST systems; IP can be secured and not actively used by larger companies, thereby blocking further development, and databases need constant and costly curation, which may present a barrier from a financial point of view. However, proper IP and data management provides competitive advantages when developed into commercially available AST tools.

### Formal regulatory aspects and landscape

Clearance by the FDA is mandatory before marketing a diagnostic device in the United States for in vitro diagnostics (IVD) purposes. In some countries, including China, FDA registration may be a requirement in addition to the local registration needs. The main target of the procedure is to qualify and quantify safety, performance, risk of misinterpretation and the benefit–risk ratio. The FDA approval process is highly formal, and manufacturers seeking FDA approval for a rapid AST system should follow the published FDA guidelines. In the European Union, AST systems are regulated by the Directive 98/79/EC (IVD directive). This is changing with the current introduction of the Regulation 2017/746 of the European Parliament and of the Council, which began in 2017. It requires design, development and manufacturing to be performed under a (still voluntary, except for Canada) quality system according to ISO 13485 (ISO is the International Organization for Standardization, which promotes the development and use of worldwide proprietary, industrial and commercial standards). In the United States, the classification of growth-based AST systems, or AST after initial growth, is well regulated. The Guidance for Industry and FDA: Class II Special Controls Guidance Document: Antimicrobial Susceptibility Test Systems (2009) was developed for the classification or re-classification of AST systems when the device is a system using short-term incubation (less than 16 h). In addition to this FDA guideline, the ISO standard 20776 adds further detail to the specifications and requirements for AST devices. A manufacturer who intends to market a device of this generic type must conform to the general controls of Section 513(a)(1)(B) of the Federal Food, Drug and Cosmetic Act, address the risks to health associated with the AST system and obtain a substantial equivalence assessment from the FDA before marketing the device. Substantial equivalence to an already commercially available device is loosely based on its intended use, the qualities of its design, the nature of the materials from which it is built, analytical performance, the safety of the user, effectiveness and some other even less strictly defined characteristics. The FDA believes that the performance of such a device can be best mapped by comparing it with the CLSI reference methods^22–24^.

Regulatory aspects can provide barriers to AST development, as the clinical validation and trials are lengthy and costly, often too costly to be borne by a small-sized or medium-sized enterprise. In addition, there is an absolute need for specialized knowledge, which is not easy to acquire: experienced employees with this specific expertise are hard to find. Finally, discussion between companies and regulatory bodies may be compromised by the fact that there are differences in expertise between the two entities that do not always match.

### Optimizing target product profiles

A target product profile (TPP) is a document that defines the layout and instruction for use of a commercial diagnostic test or device that will be developed into a product. A well-designed TPP directs a company to embark on a development program that is efficient and lists all important medical, technical and scientific information needed to reach an optimal commercial outcome. On the basis of a recent definition, a TPP should be developed using efforts that include information provided by scientific researchers, funders and donors, policy makers, laboratory directors and clinicians, politicians and (industrial) test developers with respect to the optimal operational and clinical characteristics for the laboratories where the tests will be used^25,26^. Usually, existing diagnostic ecosystems and diagnostic development landscapes need to be surveyed, and a TPP will vary depending on the site, the purpose and the target group intended for implementation of the test. Some important considerations concern quality control, maintenance and calibration, the ability to export data and performance-related requirements such as the TTR, sensitivity and specificity, as well as the hands-on and training time. There are desired and minimum requirements set in a TPP that should define the needs of the diagnostic stakeholders and provide a means of communication with test developers to ensure that fit-for-purpose systems are developed.

**Fig. 2:**
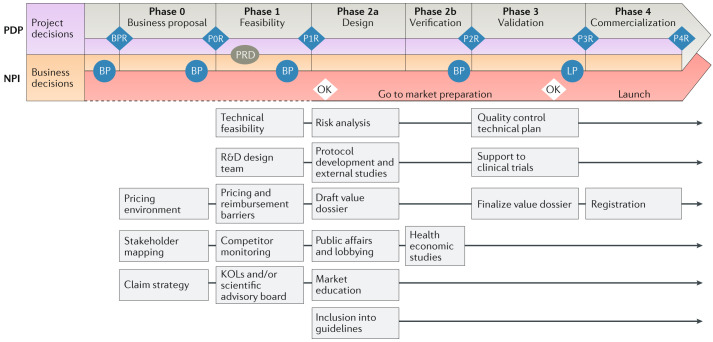
Schematic overview of the development process for products in the in vitro diagnostics market. The product development plan (PDP) consists of six distinct phases. The business proposal is composed during phase 0, usually taking results of basic research into consideration. Definition and feasibility studies are performed in phase 1, followed by design and verification in two separate phases (2a and 2b). Phase 3 involves validation, and the final phase, phase 4, comprises commercialization. To streamline this process, there is an initial business plan review (BPR) and subsequent phase reviews (P0R to P4R) after all phases. During phase 1, a product requirements document (PRD) is developed. The PRD takes into account all technical and medical needs this product should ultimately meet. The project decision may still lead to changes in the overall planning. New product introduction (NPI) is a distinct process (in parallel with the PDP) that focuses on the successful introduction of the new product. Whereas the PDP is mostly focused on technical and medical requirements, the NPI also takes customer requirements, market needs, pricing, medical–economic value and other parameters into account. For the NPI, similar reviews are planned during the process, which go straight from the business plan (BP) to launch planning (LP). At two stages, very important development decisions are taken. At the end of phase 1, an ‘OK’ is needed, as well at the end of phase 3. At these stages, labour-intense and costly further development stages will be agreed upon by the development team. The boxes below the planning schemes identify some of the most important parameters that are studied during the various stages of the PDP and NPI. Note that specific investigations and changes are continually being investigated at the various stages. The dashed line implies the possibility for a development process to still be aborted during these phases. From phase 2a onward this is much less likely to happen. KOLs, key opinion leaders; R&D, research and development.

The development of a new AST system starts with an idea and ensuing product development until final commercialization. In between are many important steps (Fig. 2) at which an extensive version of a product development scheme is presented. Not all companies will use exactly the same scheme, but the different steps shown are key in appropriate guidance of a development plan. As such, a TPP description is followed by a product development plan (PDP), which generally consists of six distinct phases. Phase 0 is when a business proposal is composed and IP issues are studied. The latter involves not only the management of proprietary IP but also the analyses of freedom to operate and competing IPs. This implies that a concept product already exists and proof of principle and proof of concept have been achieved. At the end of phase 0, a product design review (PDR) is performed whereby an independent panel of experts judges and validates the product. PDRs are managed by a design review committee consisting of experts in various domains of test development (for example, legal, business development and technology). Definition and feasibility studies are performed next (phase 1), followed by another PDR in two separate phases (2a and 2b). Phase 3 involves validation, which is then followed by a third PDR. Entry into the final phase 4 (commercialization) requires a fourth PDR. This protocol requires documentation and (long-term) data storage. New product introduction (NPI) is a distinct process (in parallel with the PDP) that focuses on the successful introduction of the new product (the ultimate target being product launch) (Fig. 2). Project risk management, safety risk management, design optimization and design transfer are just some of the obligatory processes that need to safeguard the quality of product development. Mandatory documents are the design history file, the risk management file and the design output and device master record. Obviously, product planning encompasses many stakeholders.

For all AST products, the process outlined above needs to be considered before FDA approval (Table 2). Only tests that have passed that ultimate FDA approval step can be marketed as being IVD compatible. Even though small-sized and medium-sized enterprises may offer innovation and a flexible attitude, it is considerably easier for larger diagnostics companies with the necessary critical mass and financial backing to accumulate expertise in clinical development, regulatory aspects and communication and to undertake extensive (and therefore expensive) clinical studies^27^. This is a substantial barrier to market penetration of new products developed by small companies. For these reasons, several small companies have succeeded via partnering with larger companies in the final development stages. New modes of financing preclinical validation research and sponsorship for defraying the costs of more formal development steps are therefore required. Optimal training of customers and high-class field support in case of problems are key for manufacturers.

**Table 2:** Global requirements for the development of a new AST platform before submission of the file to the FDA

General requirements	Specification
Intended use statement	• The intended use should be specified and be representative of the target populations tested with good performance characteristics • The intended use must indicate organism groups and any instrumentation the device may be used with • An example of a typical intended use statement: the ABC system is intended for the in vitro qualitative or quantitative determination of antimicrobial susceptibility of rapidly growing aerobic non-fastidious Gram-positive and Gram-negative organisms on the ABC Instrument
Summary and explanation of the test	• The summary and explanation of the test section must include whether the assay is quantitative (MIC) or qualitative (RIS) and whether results may be read and reported manually
Principle of the method	• The principles must be explained • A concise description of the technological features of the device and how the device is to be used with patient samples needs to be included
Reagents	• Antibiotics and the concentration ranges and abbreviations must be listed. These must be included in the reagent section of the labelling or on each package container if different for different devices • To prevent confusion between different drugs with similar names, abbreviations as recommended by their manufacturer should be used • The product insert should be flexible to accommodate additional antimicrobial agents • Charts should be used when possible to facilitate additions of future antimicrobial agents, limitations and performance characteristics
Directions for use	• A step by step outline of recommended procedures must be presented
Quality control	• The step by step outline of the procedure must describe quality control procedures and materials required with details of calibration • It should be ensured that the specifics of calibration and quality control are well aligned with performance claims • All recommended quality control strains and the expected results when tested with each antimicrobial agent must be defined
Reporting of results	• The interpretive criteria for each antimicrobial agent on the MIC or breakpoint device based on the FDA interpretive standards must be explained • Automated systems should have the interpretations included in the software, but if manual readings are an option, a chart of thresholds to be used for susceptibility, intermediate resistance and full resistance (RIS) interpretations must be included • Results should not be reported in instances when good performance has not been established • FDA suggests that suppression of results be software driven
Limitations	• Statements of limitations of the procedure are mandatory • If the device has software-generated interpretations, these limitations should be incorporated into the software • The following are examples: - Recommend the use of an alternative method prior to reporting of any results when the activity for any antibiotic suggests organisms with unacceptable very major error or major error rates - If not enough resistant organisms were tested, this should be mentioned. This limitation may not be necessary if a sufficient number of evaluable results close to the interpretative categories are available - If the reproducibility results for any antimicrobial agent using one procedural option are not reproducible while another option is reproducible, one should take a limitation against reporting results - An alternate method for any organism group that had a ‘no growth’ rate greater than 10% should be recommended. AST systems may be able to provide results for organisms that may not be appropriate for all of the antimicrobial agents provided on a test panel or system
Performance characteristics	• Specific performance characteristics of the assay, including the study design, stating the reference method used, and the number of sites, must be included • The percentage of essential and/or categorical agreement must be shown in table format with the reference method for each antimicrobial agent from comparative performance evaluations • Results of reproducibility studies in either a table format or a summary paragraph describing the type of study and a statement that all reproducibility results were acceptable at greater than 95% must be included

AST, antimicrobial susceptibility testing; MIC, minimum inhibitory concentration; RIS, resistant, intermediate and susceptible.

### Management and regulation of quality management systems

A quality management system (QMS) is a formalized system of policies, processes, procedures and responsibilities required for planning and execution in all areas of an organization that are in contact with the product or the customer. This includes development, marketing, manufacturing, sales, supply chain and customer service. It helps to coordinate and direct activities to continuously meet customer and regulatory requirements. Starting the implementation of a QMS early in product development with the support of outside specialized consultants will help shaping a QMS for acceptable investments.

A QMS is needed to comply with IVD regulations. Its latest possible time point for implementation is after proof-of-principle studies have been performed, when completing phase 0 (Fig. 2). This implies that after finalization of the TPP and the feasibility studies, a QMS should be in place. Otherwise, AST development will slow down, with the risk of the project being compromised (for example, by rising costs of development). QMS development will be important in the transition from basic AST research to the establishment of a routine AST platform. A good and flexible QMS system enables planned deviations from the standard development process and minimizes the re-development of academic findings. A QMS guarantees the quality control and assurance of new tests to provide optimal test quality to both the users in the diagnostic laboratories and the patients subjected to testing.

There is no way to abrogate the QMS in PDPs, as governing bodies will not accept registration efforts. We feel that there is no need for a fully implemented QMS for research in academia but that having a global knowledge of quality requirements would be helpful in preventing development without quality assessment. We emphasize that close interactions and collaborations between academic institutes and industry should be initiated very early in the diagnostic development process to ensure that scientific discovery, invention and development processes are productively coupled.

Quality assessment programmes and assessment of the impact of quality improvement strategies are essential in evaluating the performance of new AST platforms in the microbiology laboratory. Survey reports, information bulletins, correspondence, on-site consultations, educational assistance tutorials and education are used as quality improvement strategies that need to be repeated on an annual basis^28^. Improvement in QMS is strongly dependent on external assessment and education. European organizations (for example, the UK National External Quality Assessment Service (NEQAS) and Quality Control of Molecular Diagnostics (QCMD)) help to exert external comparative quality assessment in good detail^29–31^. Although regulations must be complied with, quality standards (as defined by recognized governing bodies such as, for example, the ISO, the FDA, European Commission and the European Medical Device Vigilance System (MEDDEV)) can be applied voluntarily and have been developed through a consensus process. Regulations and standards are fundamental to developing harmonized procedures to the advantage of customers and users of AST systems. A QMS integrates the elements required by regulation, standardization and guidance documents. The most important regulations in the AST development and implementation domain are issued by the FDA with their code of federal regulations^32^. In addition, the IVD directive 98/79/EC and IVD regulation 2017/746 are the main European drivers towards regulation (among others). For standardization, the ISO has an important role, issuing standards for QMSs (ISO 9001:2015), risk management (ISO 14971:2012), product labelling (ISO 18113) and stability (ISO 23640:2011), among others. Guidelines on quality management for medical devices have been issued by the European Union (MEDDEV 214/3;2007), and such devices are covered by ISO 13485:2016. The QMS should ensure appropriate data management, customer satisfaction, improvement opportunities, quality analysis and ultimately product quality. Overall this should reduce waste, lower production costs, engage staff and meet customer expectations. AST platform test qualities are defined by sensitivity, specificity and positive and negative predictive values, which are parameters that should be covered by quality assurance and external validation studies^33,34^. Importantly, the FDA has defined guidance for the use of test devices in the domain of human genomic testing, and it is anticipated that similar guidance for the molecular identification and characterization of microbial species and their phenotypes, including resistance to antimicrobials, will (have to) follow soon^35^.

### Assessment and evidence of clinical utility and validity

It is generally accepted that AST systems should always be available (that is, at any time of any given day) and should be more rapid. Results should preferably be available in less than 1 h from sampling, but a time frame within a single working shift would be a major improvement. This may depend on the actual focus of the test, and the medical–economic value of new AST platforms should be substantial. It has been claimed that the results of diagnostic microbiology testing affect clinical therapy decision making for about 50% of patients, whereas results generating changes from empiric to more targeted antibiotic therapy involve 34% to 56% of patients^36^. Assuming that bacterial identification has been already performed and is not a limiting factor, paediatric infectious disease specialists seem to be the most frequent users of AST services, whereas emergency room doctors request the smallest number of such tests. However, the availability of and access to accurate and rapid AST will not always result in increased use of either the service or the data generated. According to recent market feedback, both inpatients and outpatients are not willing to delay their first dose of antimicrobials for 15 min and even less are willing to delay for 30 min (G.L., unpublished observations). Therefore, rapid AST systems will mostly have a role in the switch to better defined therapy^37–39^. Still, to implement rapid AST systems, we need better delivery of diagnostic care to provide better clinical care at the same time. Even so, generating accurate susceptibility profiles before the second dose of empirical therapy would offer major advances in the treatment of bacterial infections and antibiotic stewardship. Clinical trials and behavioural change, to be defined for different hospitals with sometimes large differences in organization, may be required to determine the actual added value of waiting up to 1 h (‘watchful waiting’ approach) for an AST result compared with the immediate administration of an antibiotic. It has to be emphasized that detection of possibly small but major differences in terms of value added may need large and costly clinical studies. Nevertheless, it seems obvious that the decision between immediate treatment versus waiting for a susceptibility profile and more targeted treatment should be based on the (perceived) urgency of the clinical situation of the patient. Finally, all efforts into rapid AST are useless if pre-analytical factors (including sample transport) are neglected. The urgency for rapid AST will automatically increase with the emergence of more multidrug-resistant bacterial species.

### Cost-effectiveness and health economics studies

The cost of consumables accompanying traditional microbial identification and AST (such as agar plates, liquid media and commercial diagnostic systems) has always been relatively modest, with the main budget expense in a clinical microbiology laboratory being cost of labour^40^. AST results are often not released from the instrument for hours. Currently, the value of the introduction of expensive diagnostics has been quite selectively studied during the implementation of nucleic acid-based quantitative detection, whereby the test is offered to only a well-defined patient population (such as patients infected with HIV or hepatitis C virus). The availability of a somewhat costly direct-from-blood, nucleic acid-based detection platform for microbial pathogens and resistance markers did not immediately show its medical value to the individual patient^41^. However, the Roche SeptiFast assay and the Abbott Iridica test^42^ were never broadly implemented and thus were unsuccessful; both were too expensive to be made available to all patients, and their uptake in the diagnostic market failed. The systems were never made available to the US market, so it is not known whether they might have done better in the American medical ecosystem. Still, the characteristics of the two tests and their performance relative to existing diagnostics were not viewed as justification for their proposed clinical benefit. On the basis of theoretical assumptions, probably fewer than 20–25% of all patients may benefit from such add-on diagnostic tools^41^. Still, negative test results and the evidence for discontinuation of treatment will ultimately have a distinct value only if a satisfactory negative predictive value of the test can be demonstrated. Relatively expensive tests in health-care economies that require the patient or an insurer to pay for or reimburse the cost of a diagnostic test may not represent good odds. Without the preselection of high-risk patients, any added value will be diluted by the large number of patients who will not gain benefit from the new platform. At the level of an individual hospital laboratory, which very often has a limited budget, it is hard to demonstrate the relevance of possible savings following the implementation of new microbial diagnostic platforms. The real impact and cost savings incurred by new AST platforms are more likely to be felt in actual clinical departments rather than in the laboratory, hence in a budget silo where the primary costs for testing are not actually paid for. The difference in speed between many of these systems and an accelerated conventional phenotypic test (disk diffusion or a gradient test) recalibrated to shorter incubation time (4–8 h) is only very small or non-existent, and the question remains whether this correlates with improved clinical effectiveness. The successful implementation of relatively expensive diagnostic systems will require prospective clinical outcome studies, as well as the development of personalized diagnostics approaches. Personalized diagnostics will require an upfront risk assessment to select high-risk patient cohorts; the general concept of individual risk assessment has already been accepted by the clinical community. However, the value to patient management and the outcome of a systematic risk assessment, as an upfront gatekeeper for access to relatively expensive diagnostic assays, has not yet been evaluated. There is a global need for integrative systems-based practices covering clinical care, diagnostics, treatment and intelligent antibiotic stewardship. Improvement in availability and use of electronic medical records (EMR) would be an important step in the right direction.

Risk assessments based on machine learning or causal probabilistic network analyses have been applied to population-based predictions of major outbreaks of infection^43^, as well as in the risk assessment of individual patients^44^. A new algorithm was able to select patients at a higher risk of systemic infection. Future clinical outcome studies may identify other potential risk factors associated with predicting the individual risk (or likelihood of risk) of infected patients without the use of expensive add-on diagnostics that may provide faster AST and thus an improved clinical outcome. A detailed comparison between clinical scenarios in developed and developing economies is required. Funding models around insurance and reimbursement should be made more attractive and go beyond the classic governmental research incentive. Specific funds for development, industrialization and product design should be made available, which would promote the uptake of innovative AST strategies.

## Applications

### Antimicrobial susceptibility testing and new antibiotics

AST and AMR surveillance are important parts of the development of new antibiotics, as infection prevention and infectious disease practitioners will not generally introduce a new antibiotic into health care until they understand its use^45^. Furthermore, AST data and proposed breakpoints for new antibiotics have to be submitted as part of regulatory approval procedures. Investing in AST during antibiotic development could help reduce the costs of clinical trials^46^. Co-development of new antimicrobials together with specific AST for target microorganisms may help to better position an antibiotic in the clinical market^47^. In an era of pan-drug resistance, we are reconsidering empiric broad-spectrum antibiotic-prescribing policies, and the identification of bacterial resistance to novel antimicrobials using AST may not necessarily mean that an already available, or previously ‘shelved’, antibiotic needs to be discarded but that it needs to be considered in alternate prescribing schemes such as antibiotic mixing or cycling^48^. Novel and rapid AST should be versatile and adaptable to changes in the way in which (new) antibiotics are prescribed, both currently and in the future.

### The institutional antibiogram

As a means to drive the use of AST data as part of the accreditation process for clinical microbiology laboratories leading to the certification by the Clinical Laboratory Improvement Amendments (CLIA), the College of American Pathologists (CAP) publishes a checklist of guidelines that includes a call for the construction of an annual institutional cumulative antibiogram to assess local AMR trends. Specifically, MIC.21946 mandates that certified laboratories maintain cumulative AST data and report these to medical staff at least once a year. CLSI has developed guidelines (the most recent being M39-A4)^49^ to provide a standardized template for the preparation of institutional antibiograms^50^. An effort to generate data that can be compared between reporting periods and between institutions means that the rules for this guideline have become fairly complex, although the basic principles of M39-A4 remain, including an at least annual cumulative antibiogram report that contains only verified final results. The cumulative data should include antibiotics that are routinely used by the reporting institution. However, even in this format, it should be noted that the yearly institutional antibiogram might not provide feedback that is sensitive enough to measure the effectiveness of antimicrobial stewardship interventions but could provide advice when a unit-specific antibiogram is used^51^. The institutional antibiogram functions as a guide for automatically selecting the correct therapy for infections in cases when the causative microorganism has been identified^50^. Two recent publications have used existing institutional antibiograms to develop a Weighted-Incidence Syndromic Combination Antibiogram (WISCA)^52,53^. The authors considered the effectiveness of empiric antibiotic prescribing for monomicrobial or polymicrobial infections before AST results had even been generated. They used information (specific organism–antibiotic combination effectiveness assumptions) gained from traditional antibiograms as a guide.

An institutional antibiogram may be integrated in machine learning-based software. Machine learning is used for the development of explicit algorithms with predictive power and is closely related to computational statistics. Within the field of data analytics, machine learning is a method used to design complex models that lend themselves to prediction. Adoption of these models will enable AST researchers to suggest reliable decisions and uncover new insights through learning from historical antibiotic resistance data trends. Such decision-support software programmes may provide guidance for optimal empirical antimicrobial treatment on the basis of available clinical and laboratory information in the electronic patient record and information on local microbial flora and AMR profiles^44^.

### The ‘smart antibiogram’

Although the institutional antibiogram is a powerful tool, it will never eliminate the need for new and improved AST systems. The current frequency at which these antibiograms are updated may mean that they are not quickly adaptable to rapidly changing or emerging antibiotic resistance mechanisms that occur within a particular health-care institution^54^. If we consider recent and future technological advances in the rapid identification of the causative agent, for example, the use of molecular assays or MALDI-TOF-MS to detect microorganisms directly from positive blood culture bottles, then a more refined system for susceptibility (or resistance) could be generated via a ‘smart antibiogram’. First, the smart antibiogram has to have real-time access to rapid cumulative antibiotic profiling data and be essentially self-updating with all the appropriate rules and logic in place to aggregate relevant antibiotic profiling data. Again, in this case, accessible EMRs would be important, although there may be issues with non-discrete microbiological data and rules. A smart antibiogram system must also have the flexibility to identify trends or parameters on the basis of increases in individual antibiotic MICs via the addition of multidrug resistance rules and alerts to the algorithm used. Second, by limiting the number of isolates recorded per patient to the initial isolate only, the M39-A4 guidelines prevent extrapolation of resistance profiles. If the same multidrug-resistant microorganism was recovered from the blood, urine and respiratory secretions of a single patient yet only the blood isolate was represented in the cumulative antibiogram, the data would not reflect the overall susceptibility of all isolates recovered from urine or respiratory tract specimens. Further, this procedure does not enable antimicrobial stewardship or infection prevention personnel to observe the accumulation of antibiotic resistances in patient isolates during the course of antibiotic therapy.

Using AST data from single patient isolates recovered from different sources would enable search parameters to include the selection of both specimen type^55–60^ and specific organisms^59–61^, thereby providing more realistic algorithms to guide empiric treatment. Antimicrobial prescription and institutional antibiograms differ between various institutions, sometimes even within an individual health-care institution^60,62,63^, and between inpatient and outpatient populations^60,63–65^. They may change on the basis of length of hospital stay^66^ and may depend on the age of the patient^65,67^. In addition, more specific attributes of the causative microorganism, for example, the presence of antibiotic-resistant populations^61^ or whether an organism was recovered through surveillance culture or from a diagnostic specimen^64^, could be recorded. Therefore, the smart antibiogram should be developed to use a whole range of search parameters and patient demographics in its calculations. This will facilitate the automatic fine tuning of smart antibiogram algorithms and increase their value in predicting the correct empiric antibiotic therapy for individual microorganisms detected in different clinical specimens at different times on different wards and from different populations. For that, statistical approaches must be developed that would help predict correct antibiotic treatment on the basis of prior experience and the use of intelligent databases.

## Concluding statements

AST is an important part of the targeted antibiotic-prescribing process for bacterial diseases, helping to cope with and reduce the growing emergence and spread of AMR, informing on the success of infection control measures and ultimately saving the lives of patients. However, there still remains a need for novel and rapid AST diagnostics to be developed and implemented, which is not always appreciated by clinicians and health-care authorities. For example, it seems logical that appropriate AST approaches should be considered a major factor for monitoring and responding to infectious disease outbreaks. Yet, even recent publications^68^ tend to pay little attention to the need for AST in helping limit and prevent outbreaks. Better communication about the importance of AST should lead to better collaboration between the public, academia, patient groups, policy makers and industry. Understanding AST platform development and implementation issues will lead to a better understanding of the barriers and solutions required by public and private entities in maximizing the availability and use of (new and rapid) AST platforms. The AST platforms that are currently available are robust and represent added value to the clinical diagnostic microbiology laboratory, but their main shortcoming is the somewhat long TTR and lack of automation. The TTR of traditional AST is being reduced from 16–20 h to 4–8 h but requires recalibration when doing so, including, for example, AST tests that generate MICs. New AST platforms have been developed and proposed, but there is a lack of market penetration and, hence, further innovation. In this Consensus Statement, we presented barriers and possible solutions to the development and implementation of AST in health-care settings, ultimately resulting in the potential use of institutional smart antibiograms. We hope that this knowledge will lead to increased dialogue and understanding between AST developers and end users, leading to a positive impact on patient care.

## Supplementary information

Supplementary information S1

## References

[1] European Centre for Disease prevention and Control (ECDC), European Medicine Agency (EMEA). *The bacterial challenge: time to react* (ECDC, 2009).

[2] Llor C (2017). The STOP-AB trial protocol: efficacy and safety of discontinuing patient antibiotic treatment when physicians no longer consider it necessary. BMJ Open.

[3] Cangelosi GA, Meschke JS (2014). Dead or alive: molecular assessment of microbial viability. Appl. Environ. Microbiol..

[4] Nault V (2017). Sustained impact of a computer-assisted antimicrobial stewardship intervention on antimicrobial use and length of stay. J. Antimicrob. Chemother..

[5] Holcomb ZE, Tsalik EL, Woods CW, McClain MT (2017). Host-based peripheral blood gene expression analysis for diagnosis of infectious diseases. J. Clin. Microbiol..

[6] Van Belkum A, Dunne WM (2013). Next-generation antimicrobial susceptibility testing. J. Clin. Microbiol..

[7] Pulido MR, García-Quintanilla M, Martín-Peña R, Cisneros JM, McConnell MJ (2013). Progress on the development of rapid methods for antimicrobial susceptibility testing. J. Antimicrob. Chemother..

[8] Liu VX (2017). The timing of early antibiotics and hospital mortality in sepsis. Am. J. Respir. Crit. Care Med..

[9] Van der Eijk AA, Tintu AN, Hays JP (2017). Pre-implementation guidelines for infectious disease point-of-care testing in medical institutions. Future Microbiol..

[10] Idelevich EA (2014). Acceleration of antimicrobial susceptibility testing of positive blood cultures by inoculation of Vitek 2 cards with briefly incubated solid medium cultures. J. Clin. Microbiol..

[11] Liu YY (2016). Emergence of plasmid-mediated colistin resistance mechanism MCR-1 in animals and human beings in China: a microbiological and molecular biological study. Lancet Infect. Dis..

[12] Lübbert C (2017). Environmental pollution with antimicrobial agents from bulk drug manufacturing industries in Hyderabad, South India, is associated with dissemination of extended-spectrum β-lactamase and carbapenemase-producing pathogens. Infection.

[13] World Health Organization Library Cataloguing-in-Publication Data. *Global action plan on antimicrobial resistance* (WHO, 2015).

[14] Review on antimicrobial resistance. *Tackling drug-resistant infections globally: final report and recommendations* (AMR, 2016).

[15] Prasad R, Bandyopadhyay TK (2014). Nanotechnology patents in the automotive industry (a quantitative and qualitative analysis). Recent Pat. Nanotechnol.

[16] Lin JC, Fan CT, Liao CC, Chen YS (2018). Taiwan Biobank: making cross-database convergence possible in the Big Data era. Gigascience.

[17] Papp-Wallace KM, Bonomo RA (2016). New β-lactamase inhibitors in the clinic. Infect. Dis. Clin. North Am..

[18] Gupta SK (2014). ARG-ANNOT, a new bioinformatic tool to discover antimicrobial resistance genes in bacterial genomes. Antimicrob. Agents Chemother..

[19] Alleweldt F (2017). Developing a framework to assess the cost effectiveness of COMPARE - a global platform for the exchange of sequence-based pathogen data. Rev. Sci. Tech..

[20] Aarestrup FM, Koopmans MG (2016). Sharing data for global infectious disease surveillance and outbreak detection. Trends Microbiol..

[21] Aarestrup FM (2012). Integrating genome-based informatics to modernize global disease monitoring, information sharing, and response. Emerg. Infect. Dis..

[22] Ferraro, M. J. & Jorgensen, J. H. in *Manual of Clinical Microbiology* (eds Murray, P. R. et al.) 1593–1600 (American Society of Microbiology, 1999).

[23] Clinical and Laboratory Standards Institute. *M07. Methods for Dilution Antimicrobial Susceptibility Tests for Bacteria that Grow Aerobically* (CLSI, 2018).

[24] Clinical and Laboratory Standards Institute. *M11-A8. Methods for Antimicrobial Susceptibility Testing of Anaerobic Bacteria* (CLSI, 2012). 31339681

[25] Denkinger CM (2015). Target product profile of a molecular drug-susceptibility test for use in microscopy centers. J. Infect. Dis..

[26] Dittrich S (2016). Target Product Profile for a diagnostic assay to differentiate between bacterial and non-bacterial infections and reduce antimicrobial overuse in resource-limited settings: an expert consensus. PLOS ONE.

[27] World Health Organization European Observatory on Health Systems and Policies. *Ensuring Innovation in diagnostics for bacterial infection: implications for policy* Ch. 12 (eds Morel, C. et al.) (WHO, 2016). 28806042

[28] Richardson H, Wood D, Whitby J, Lannigan R, Fleming C (1996). Quality improvement of diagnostic microbiology through a peer-group proficiency assessment program. A 20-year experience in Ontario. Microbiol. Committee. Arch. Pathol. Lab. Med..

[29] Van Belkum A, Niesters HG, MacKay WG, van Leeuwen WB (2007). Quality control of direct molecular diagnostics for methicillin-resistant *Staphylococcus aureus*. J. Clin. Microbiol..

[30] Te Witt R, van Belkum A, MacKay WG, Wallace PS, van Leeuwen WB (2010). External quality assessment of the molecular diagnostics and genotyping of meticillin-resistant. Staphylococcus aureus. Eur. J. Clin. Microbiol. Infect. Dis..

[31] Moran-Gilad J (2015). Proficiency testing for bacterial whole genome sequencing: an end-user survey of current capabilities, requirements and priorities. BMC Infect. Dis.

[32] Caliendo AM, Hanson KE (2016). Point-Counterpoint: The FDA has a role in regulation of laboratory-developed tests. J. Clin. Microbiol..

[33] Ieven M, Finch R, van Belkum A (2013). European quality clearance of new microbiological diagnostics. Clin. Microbiol. Infect..

[34] Grys TE (2011). Developing a quality system for quantitative laboratory-developed tests. Clin. Microbiol. Infect..

[35] Evans BJ, Burke W, Jarvik GP (2015). The FDA and genomic tests — getting regulation right. N. Engl. J. Med..

[36] AdvaMed. *The Value of Diagnostics Innovation, Adoption and Diffusion into Health Care* (The Lewin Group, Inc., 2005).

[37] Kiehlbauch J, Kendle JM, Carlson LG, Schoenknecht FD, Plorde JJ (1989). Automated antibiotic susceptibility testing: comparative evaluation of four commercial systems and present state. Clin. Lab. Med..

[38] Li B, Qiu Y, Shi H, Yin H (2016). The importance of lag time extension in determining bacterial resistance to antibiotics. Analyst.

[39] Syal K (2017). Current and emerging techniques for antibiotic susceptibility tests. Theranostics.

[40] Brezmes MF, Ochoa C, Eiros JM (2002). Cost analysis in a clinical microbiology laboratory. Eur. J. Clin. Microbiol. Infect. Dis..

[41] Westh H (2009). Multiplex real-time PCR and blood culture for identification of bloodstream pathogens in patients with suspected sepsis. Clin. Microbiol. Infect..

[42] Jordana-Lluch E (2015). Evaluation of the broad-range PCR/ESI-MS technology in blood specimens for the molecular diagnosis of bloodstream infections. PLOS ONE.

[43] Jafarpour N, Izadi M, Precup D, Buckeridge DL (2015). Quantifying the determinants of outbreak detection performance through simulation and machine learning. J. Biomed. Inform..

[44] Paul M (2016). Prediction of bacteremia using TREAT, a computerized decision-support system. Clin. Infect. Dis..

[45] De With K (2016). Strategies to enhance rational use of antibiotics in hospital: a guideline by the German Society for Infectious Diseases. Infection.

[46] O’Dwyer K (2015). Bacterial resistance to leucyl-tRNA synthetase inhibitor GSK2251052 develops during treatment of complicated urinary tract infections. Antimicrob. Agents Chemother..

[47] Spellberg B (2014). The future of antibiotics. Crit. Care.

[48] Van Duijn PJ (2018). The effects of antibiotic cycling and mixing on antibiotic resistance in intensive care units: a cluster-randomised crossover trial. Lancet Infect. Dis..

[49] Clinical and Laboratory Standards Institute. *M39-A4. Analysis and presentation of cumulative antimicrobial susceptibility test data* (CLSI, 2014).

[50] Hindler JF, Stelling J (2007). Analysis and presentation of cumulative antibiograms: a new consensus guideline from the Clinical and Laboratory Standards Institute. Clin. Infect. Dis..

[51] Schulz LT, Fox BC, Polk RE (2012). Can the antibiogram be used to assess microbiological outcomes after antimicrobial stewardship interventions? A critical review of the literature. Pharmacotherapy.

[52] Hebert C (2012). Demonstration of the weighted-incidence syndromic combination antibiogram: an empiric prescribing decision aid. Infect. Control Hosp. Epidemiol..

[53] Randhawa V (2014). Weighted-incidence syndromic combination antibiograms to guide empiric treatment of critical care infections: a retrospective cohort study. Crit. Care.

[54] Tibbetts R, Frye JG, Marschall G, Warren D, Dunne W (2008). Detection of KPC-2 in a clinical isolate of *Proteus mirabilis* and first reported description of carbapenemase resistance caused by a KPC β-lactamase in *P. mirabilis*. J. Clin. Microbiol..

[55] Etani T (2017). Antimicrobial susceptibility of pathogens in acute uncomplicated cystitis cases in the urology department of a community hospital in Japan: comparison with treatment outcome and hospital-wide antibiogram. J. Infect. Chemother..

[56] Chidester JR (2016). Antibiogram for periprosthetic infections: a tool for better informed selection of empiric antibiotics for surgical site infections. Ann. Plast. Surg..

[57] Gangcuangco LM, Alehandria M, Henson KE, Saniel M (2016). Antimicrobial susceptibility of *Escherichia coli* in uncomplicated cystitis in the emergency department: is the hospital antibiogram an effective treatment guide?. Acad. Emerg. Med..

[58] Rabs N, Wieczorkiewicz SM, Costello M, Zamfirova I (2014). Development of a urinary-specific antibiogram from gram-negative isolates: impact of patient risk factors on susceptibility. Am. J. Infect. Control.

[59] Smith ZR (2016). Development of a combination antibiogram for *Pseudomonas aeruginosa* bacteremia in an oncology population. J. Oncol. Pharm. Practice.

[60] Smith SC, Bazzoli C, Chung I, Johnson A, Martin DR (2015). Antimicrobial susceptibility of *Escherichia coli* in uncomplicated cystitis in the emergency department: is the hospital antibiogram an effective treatment guide?. Acad. Emerg. Med..

[61] Hsu AJ (2015). The use of a combination antibiogram to assist with the selection of appropriate antimicrobial therapy for carbapenemase-producing *Enterobacteriaceae* infections. Infect. Control Hosp. Epidemiol..

[62] Hill JN, Suda KJ, Ramanathan S, Evans CT (2015). Development of a unit-specific antibiogram and planning for implementation: pre-implementation findings. Am. J. Infect. Control..

[63] Hines MC (2015). Resistance patterns of *Escherichia coli* in women with uncomplicated urinary tract infection do not correlate with emergency department antibiogram. J. Emerg. Med..

[64] Gray KL, Fulcher LC, McElmeel ML, Xenakis EM, Jorgensen JH (2011). The outpatient institutional antibiogram does not accurately reflect the susceptibility of prepartum group B streptococcal isolates to erythromycin and clindamycin. Diagn. Microbiol. Infect. Dis..

[65] Dahle KW, Korgenski EK, Hersh AL, Srivastava R, Gesteland PH (2012). Clinical value of an ambulatory-based antibiogram for uropathogens in children. J. Ped. Infect. Dis..

[66] Anderson DJ, Miller B, Marfatia R, Drew R (2012). Ability of an antibiogram to predict *Pseudomonas aeruginosa* susceptibility to targeted antimicrobials based on hospital day of isolation. Infect. Control Hosp. Epidemiol..

[67] Ventura MM, Brittain K, Pruskowski J, Hogan D, Walker T (2015). Development of an age-dependent antibiogram in a veterans affairs community. J. Am. Geriatr. Soc..

[68] Perkins MD (2017). Diagnostic preparedness for infectious disease outbreaks. Lancet.

[69] Kaman WE, Elshout G, Bindels PJ, Mitsakakis K, Hays JP (2016). Current problems associated with the microbiological point-of-care testing of respiratory tract infections in primary care. Future Microbiol..

[70] Steingart KR (2013). Xpert® MTB/RIF assay for pulmonary tuberculosis and rifampicin resistance in adults. Cochrane Database Syst. Rev..

[71] Gibson J (2017). Multi-center evaluation of the cobas® Liat® Influenza A/B and RSV assay for rapid point of care diagnosis. J. Clin. Virol..

[72] Schnee SV, Pfeil J, Ihling CM, Tabatabai J, Schnitzler P (2017). Performance of the Alere i RSV assay for point-of-care detection of respiratory syncytial virus in children. BMC Infect. Dis.

[73] Nijhuis RHT, Guerendiain D, Claas ECJ, Templeton KE (2017). Comparison of ePlex respiratory pathogen panel with laboratory-developed real-time PCR assays for detection of respiratory pathogens. J. Clin. Microbiol..

[74] Turner KM (2017). Analysis of the potential for point-of-care test to enable individualised treatment of infections caused by antimicrobial-resistant and susceptible strains of *Neisseria gonorrhoeae*: a modelling study. BMJ Open.

[75] Chakravorty S (2016). Detection of isoniazid-, fluoroquinolone-, amikacin-, and kanamycin-resistant tuberculosis in an automated, multiplexed 10-color assay suitable for point-of-care use. J. Clin. Microbiol..

[76] Chakravorty S (2017). New Xpert MTB/RIF Ultra: improving detection of *Mycobacterium tuberculosis* and resistance to rifampin in an assay suitable for point-of-care testing. mBio.

[77] Bongard E (2015). Analytic laboratory performance of a point of care urine culture kit for diagnosis and antibiotic susceptibility testing. Eur. J. Clin. Microbiol. Infect. Dis..

[78] Mabey D, Peeling RW, Ustianowski A, Perkins MD (2004). Tropical infectious diseases: diagnostics for the developing world. Nat. Rev. Microbiol..

[79] Kettler, H., White, K. & Hawkes, S. *Mapping the landscape of diagnostics for sexually transmitted infections* (WHO/TDR, 2004).

[80] Holm A (2017). Effect of point of care susceptibility testing in general practice on appropriate prescription of antibiotics for patients with uncomplicated urinary tract infection: a diagnostic randomised controlled trial. BMJ Open.

[81] Okeke IN (2011). Diagnostics as essential tools for containing antibacterial resistance. Drug Resist. Updat..

