# Supplementary material for: Developmental roadmap for antimicrobial susceptibility testing systems
Source: Nat Rev Microbiol. 2018 Oct 17;17(1):51–62. doi: 10.1038/s41579-018-0098-9 (PMC7138758; doi:10.1038/s41579-018-0098-9)
Supplement: Supplementary file 1 — Supplementary information S1 [file 41579_2018_98_MOESM1_ESM.pdf]

nature REVIEWS MICROBIOLOGY

SUPPLEMENTARY INFORMATION
In format as provided by the authors

Developmental roadmap for antimicrobial susceptibility testing systems

Alex van Belkum, Till T. Bachmann, Gerd Lüdke, Jan Gorm Lisby, Gunnar Kahlmeter, Allan Mohess, Karsten Becker, John P. Hays, Neil Woodford, Konstantinos Mitsakakis, Jacob Moran-Gilad, Jordi Vila, Harald Peter, John H. Rex and Wm. Michael Dunne Jr, the JPIAMR AMR-RDT Working Group on Antimicrobial Resistance and Rapid Diagnostic Testing

https://doi.org/10.1038/s41579-018-0098-9

Supplementary information S1

This is an initiative of the Transnational Working Group Rapid Diagnostics funded by the Joint Programming Initiative on Antimicrobial Resistance

The JPIAMR Working Group on AMR-RDT:

Till T. Bachmann (coordinator)	University of Edinburgh	UK
Alex van Belkum	BioMérieux	FR
Alasdair MacGowan	North Bristol NHS Trust	UK
Aman Russom	KTH Royal Institute of Technology	SE
Andrew Shepherd	Omega Diagnostics	UK
Ann Van den Bruel	NIHR Diagnostic Evidence Cooperative	UK
Annika Eriksson	HemoCue AB	SE
Barbara Fallowfield	British In Vitro Diagnostics Association	UK
Cassandra Kelly-Cirino	Foundation for Innovative New Diagnostics	CH
Carla Deakin	NICE	UK
Eiichi Tamiya	Osaka University	JP
Francis Moussy	World Health Organization, *Observer*	CH
Franck Molina	European Diagnostics Cluster Alliance	FR
Frank Apostel	R-Biopharm	DE
Frank Bier	Fraunhofer IZI-BB	DE
Gerd Luedke	Curetis GmbH	DE
Guido Werner	Robert Koch Institute	DE
Gunnar S. Simonsen	University of Tromsø	NO
Gyorgy Abel	Lahey Hospital, Harvard University	US
Herman Goossens	Antwerp University	BE
Jacob Moran-Gilad	Ben-Gurion University & Ministry of Health	IL
James Fraser	Chipcare	CA
Jean François de Lavison	Ahimsa Fund	FR
John Hays	Erasmus University Medical Center	NL
John Rex	F2G, Ltd.	US
Jordi Vila	Institute of Global Health Barcelona	ES
Karsten Becker	University Hospital Münster, DGHM	DE
Kate Templeton	NHS Lothian	UK
Kirsten Miller-Duys	Hyrax Biosciences	SA
Konstantinos Mitsakakis	Hahn-Schickard	DE
Manica Balasegeram	GARDP/DNDI	CH
Mark Woolhouse	University of Edinburgh	UK
Neil Butler	Spectromics	UK
Neil Woodford	Public Health England	UK

Paul Savelkoul	Maastricht University	NL
Petra Gastmeier	Charite Belin, Infect Control 2020	DE
Philippe Lagace-Wiens	University of Manitoba (left)	CA
Ramanan Laxminarayan	Center for Disease Dynamics, Economics & Policy	US/ IN
Rosanna Peeling	London School of Hygiene &Tropical Med.	UK
Saturnino Luz	Usher Institute	UK
Sören Schubert	Max von Pettenkofer Institute Munich	DE
Stephan Harbarth	University of Geneva	CH
Sue Hill	NHS England	UK
Tracy Merlin	University of Adelaide	AU
Taslimarif Saiyed	Centre for Cellular and Molecular Platforms	IN
Thomas Wichelhaus	University Frankfurt; Paul Ehrlich Society	DE
Tjeerd van Staa	Farr Institute Health Informatics Research	UK
Valentina Di Gregori	San Pier Damiano Hospital Faenza (ex Univ of Bologna)	IT
Wouter van der Wijngaart	KTH Royal Institute of Technology	SE
Wilfried von Eiff	HHL Leipzig	DE
